# Supplementary material for: Catalysis of Organic Pollutants Abatement Based on Pt-Decorated Ag@Cu2O Heterostructures
Source: Molecules. 2019 Jul 26;24(15):2721. doi: 10.3390/molecules24152721 (PMC6696002; doi:10.3390/molecules24152721)
Supplement: Supplementary file 1 [file molecules-24-02721-s001.pdf]

Supplementary Materials

Catalysis of Organic Pollutants Abatement Based on Pt-Decorated Ag@Cu_2O Heterostructures

Xiaolong Zhang [1,2], Bingbing Han [1,3], Yaxin Wang [1,2], Yang Liu [1,2], Lei Chen [1,3,*] and Yongjun Zhang [1,2,*]

[1] Key Laboratory of Functional Materials Physics and Chemistry of the Ministry of Education, Jilin Normal University, Changchun 130103, China

[2] National Demonstration Center for Experimental Physics Education, Jilin Normal University, Siping 136000, China.

[3] College of Chemistry, Jilin Normal University, Siping 136000, China

* Correspondence: chenlei@jlnu.edu.cn (L. C.); yjzhang@jlnu.edu.cn (Y.Z.); Tel.: +86-0434-3294566 (L.C.); +86-0434-3294566 (Y.Z.)

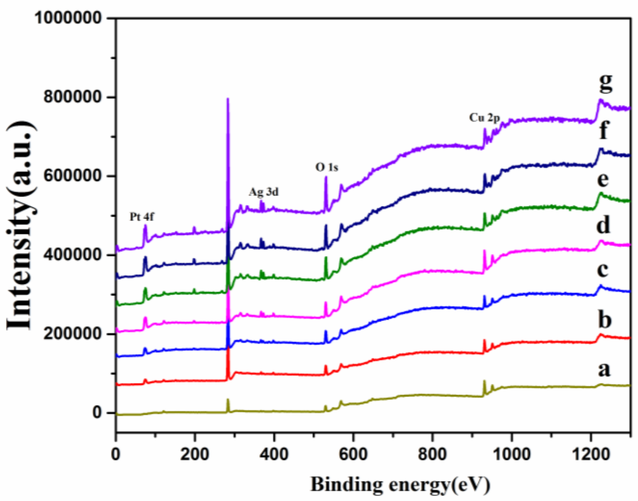

Figure S1. XPS spectra survey spectra of Ag@Cu_2O (a) and Ag@Cu_2O-Pt (b: 0.95 × 10^{-4}, c: 1.26 × 10^{-4}, d: 1.43 × 10^{-4}, e: 1.52 × 10^{-4}, f: 1.58 × 10^{-4}, and g: 1.63 × 10^{-4} mol/L).

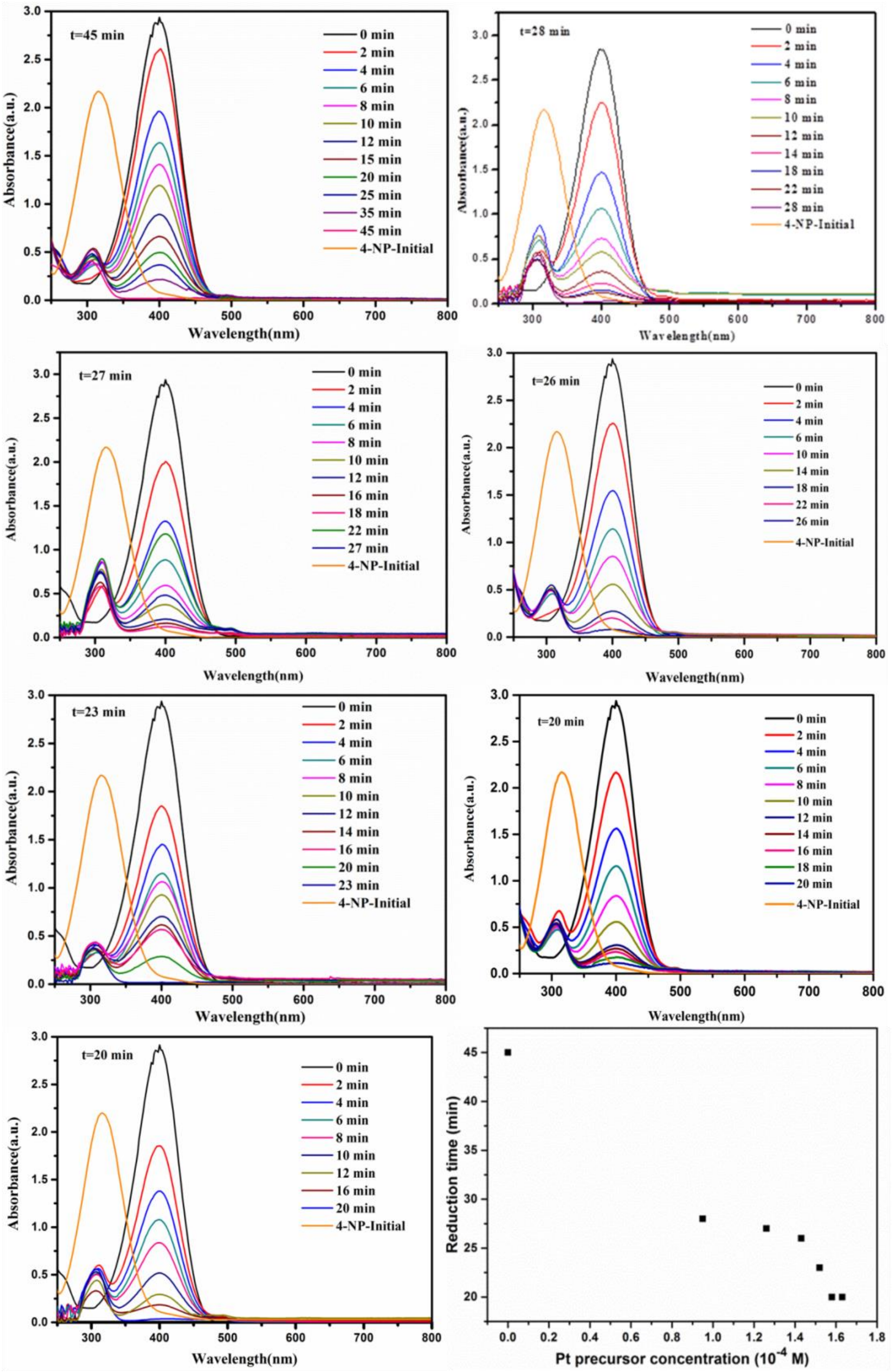

Figure S2. UV-Vis absorbance spectra of the reduction of 4-NP by $NaBH_4$ in the presence of different Ag@Cu_2O-Pt (the Pt precursor concentrations were 0, 0.95 × 10^{-4}, 1.26 × 10^{-4}, 1.43 × 10^{-4}, 1.52 × 10^{-4}, 1.58 × 10^{-4}, and 1.63 × 10^{-4} mol/L), and Catalytic reduction time vs. different Ag@Cu_2O-Pt (Pt precursor concentrations are 0, 0.95 × 10^{-4}, 1.26 × 10^{-4}, 1.43 × 10^{-4}, 1.52 × 10^{-4}, 1.58 × 10^{-4}, and 1.63 × 10^{-4} mol/L).
